# Magnetic Bead—Magic Bullet

**DOI:** 10.3390/mi7020021

**Published:** 2016-01-28

**Authors:** Christine Ruffert

**Affiliations:** Center for Production Technology, Leibniz Universitaet Hannover, An der Universitaet 2, D-30823 Garbsen, Germany; christineruffert@t-online.de; Tel.: +49-1767-6219-875

**Keywords:** bioanalysis, detection, lab-on-a-chip, magnetic bead, microfluidics, nanomedicine, nanoparticle, separation, singleplexing, superparamagnetism

## Abstract

Microfluidics is assumed to be one of the leading and most promising areas of research since the early 1990s. In microfluidic systems, small spherical magnetic particles with superparamagnetic properties, called magnetic beads, play an important role in the design of innovative methods and tools, especially in bioanalysis and medical sciences. The intention of this review paper is to address main aspects from the state-of-the-art in the area of magnetic bead research, while demonstrating the broad variety of applications and the huge potential to solve fundamental biological and medical problems in the fields of diagnostics and therapy. Basic issues and demands related to the fabrication of magnetic particles and physical properties of nanosize magnets are discussed in Section 2. Of main interest are the control and adjustment of the nanoparticles’ properties and the availability of adequate approaches for particle detection via their magnetic field. Section 3 presents an overview of magnetic bead applications in nanomedicine. In Section 4, practical aspects of sample manipulation and separation employing magnetic beads are described. Finally, the benefits related to the use of magnetic bead-based microfluidic systems are summarized, illustrating ongoing questions and open tasks to be solved on the way to an approaching microfluidic age.

## 1. Introduction

The introduction of a concept for Micro Total Analysis Systems (µTAS) by Manz *et al.* in 1990 initiated the development of new materials and technologies for the realization of fluidic microsystems [1]. The usual term lab-on-a-chip (LoC) is an alternative name for µTAS. The vision leading to the development of LoC devices was to provide systems featuring the dimensions of a credit card (called chip) allowing the integration of common laboratory functions within an area of only a few square centimeters. LoC devices are often considered as a class of micro-electro-mechanical systems (MEMS) and closely related to microfluidics, which describes the physics and manipulation of small amounts of fluids. Thus, in some respects LoC and microfluidic systems can be used as equivalent terms. Microfluidic operations include sample manipulation, mixing, reaction, separation, and detection. The LoC approach requires only extremely small fluid volumes (down to the nanoliter or even picoliter range), while saving reagent and sample resources and thus costs, while at the same time, proper interface structures have to be available for such small volumes and handling capabilities in combination with adequately skilled staff.

In the year 1998, Sinclair postulated: “To bead or not to bead: Applications of magnetic bead technology” and describes multifold magnetic bead applications in his review paper [2]. It should be mentioned, that despite the research activities in this area, the development of commercial LoC products is still fraught with risk and rather hesitantly followed by the industry. However, two and a half decades after the µTAS announcement, it seems that “The lab finally comes to the chip!” [3].

Parallel to the boom of the LoC device and microfluidic system development, nanomaterials and nanoparticles attracted a rising attention in the scientific world. Of particular interest is a class of magnetic materials consisting of ferromagnetic nanoparticles of a few tens or hundreds of nanometers in diameter embedded in a non-magnetic matrix, for example made of a polymer or quartz. This creates micro-size particles with superparamagnetic properties, which are commonly called magnetic beads—beads due to their spherical shape. Such superparamagnetic particles show a non-magnetic behavior, unless when exposed to a magnetizing field. A constant field causes immobilization, while a field gradient effects actuation, rotation or transport. The unique feature of magnetic beads is that samples bound to magnetic beads can be manipulated independently of any other microfluidic, biological or chemical processes by means of magnetic forces.

The large surface-to-volume ratio of the magnetic beads lends itself to a further use, *i.e.*, for chemical bonding of target molecules. This can be achieved by a specific surface modification providing ligands or an overall surface coating and is called surface functionalization. Various methods for a surface functionalization of the beads have been developed so far. In general, the functionalization comprises the modification of a solid material’s surface with respect to its physical, chemical or biological properties. This modification can be accomplished by different methods with a view to altering a wide range of characteristics of the surface, such as: roughness, hydrophilicity (affinity to water), surface charge (electrical potential), surface energy (excess energy at a material’s surface), biocompatibility (compatibility with living systems), or reactivity (tendency to undergo chemical reactions).

Especially magnetic beads have been well established as a standard tool for the specific attachment of biomolecules for detection, analysis, and quantification purposes [4,5,6,7]. Their wide use in research institutions is demonstrated by the high number of scientific publications focusing on magnetic bead applications. The publication number exceeds the level of thousands within specific technical journals and conference proceedings. For example, several 10,000 results are achieved, when typing “magnetic bead” in the search field of the publisher Springer, comprising journals like *Microsystem Technologies* and *Microfluidics Nanofluidics*, and more than 5000 results in the microfluidics journal *Lab Chip* published by the Royal Society of Chemistry [8]. Two review papers provided by Gijs *et al.* in 2004 and 2010, respectively, present in detail the state-of-the-art of magnetic bead handling at that point in time and main areas for magnetic bead applications: chemical and biological analysis, but also catalysis [4,9]. A review covering the synthesis and characteristic features of small superparamagnetic particles has been presented recently [10]. Physics and forces involved, when these particles are exposed to an external magnetic field, are presented as well as a number of applications.

This review paper comprises basic ideas and fundamental sciences involved, when magnetic particles with superparamagnetic properties are used in microfluidic systems. The manuscript provides an overview of the synthesis and challenges related to the fabrication of magnetic nanostructures and particle ensembles creating spherical-shaped microparticles for the use in microfluidic applications. Section 2 starts with basics on superparamagnetism and forces magnetic beads in a microfluidic system are exposed to. The physical properties of small magnetic particles and magnetic beads are presented. A focus is set on the adjustment of the particle size, where fabrication limits and practical aspects have to be considered at the same time. A challenge is to provide suitable detection methods and tools to measure the weak magnetic signal, generated by particles with nanoscale dimensions or ensembles of magnetic nanoparticles. Next, some selected magnetic bead applications are presented in Section 3. The focus is found in the fields of bioanalysis and biomedical sciences including nanomedicine. The use of the beads in nanomedicine is presented in some more detail. A typical use of magnetic beads in the area of bioanalysis is found in sandwich enzyme-linked immunoassays and magnetic particle-based immunoassays [11,12] where the beads serve as carrier substrate or as detection label. A rather unconventional application of functionalized magnetic beads is the manipulation of metallic nanoparticles for catalyst recovery [13]. However, an increasing interest of catalysts deposited on magnetic nanoparticles for catalyst separation in microfluidic systems was predicted by Gijs *et al.* in 2010 [9]. The use of magnetic beads in immunoassays and their application for catalyst recovery is both described Section 3. Section 4 illustrates practical aspects of the sample manipulation employing magnetic forces. The conclusion highlights unique selling points of the lab-on-a-chip approach in general and in particular the use of magic bullets—magnetic beads. Ongoing challenges are identified, which might be the bottleneck for a further industrialization of microfluidic magnetic bead systems. Finally, an outlook is given proposing new application areas.

Table 1 includes a number of references, which are part of this manuscript. The table should serve as a guide for this review paper and act as an appetizer by giving an overview of the aspects of magnetic bead synthesis and applications covered in Section 2, Section 3 and Section 4. The following three-part classification was applied for the setup of Table 1: Basics, Applications, Sensing.

**Table 1:** Overview of the scope of this paper and related literature.

Classification		Authors	Year	Reference
**Basics**	**Magnetic Bead Technology **	Sinclair	2015	[2]
		Gijs	2004	[4]
		Gijs *et al.*	2010	[9]
		Ruffert	2015	[10]
	**Nano- and Microparticle Fabrication**	Veiseh *et al.*	2010	[16]
		Park *et al.*	2007	[17]
		Zhao *et al.*	2005	[18]
		Kim *et al.*	2006	[19]
		Ito *et al.*	2005	[20]
		Jing *et al.*	2012	[22]
		Chen *et al.*	2009	[29]
		Bigall *et al.*	2011	[31]
		Zhang *et al.*	2008	[32]
**Applications**	**Separation with Magnetic Beads**	Pamme	2007	[6]
		Ruffert *et al.*	2014	[13]
		Zhang *et al.*	2008	[32]
		Miltenyi *et al.*	1990	[47]
		Zborowski *et al.*	2008	[52]
		Pamme *et al.*	2006	[53]
	**Magnetic Bead-based Immunoassays**	Tekin	2013	[7]
		Bhalla *et al.*	2013	[11]
		Ruffert *et al.*	2014	[12]
		Sivagnanam	2010	[39]
	**Biomedical Bead Applications**	Thanh	2012	[21]
		Colombo *et al.*	2012	[23]
		Llandro *et al.*	2010	[28]
		Figuerola *et al.*	2010	[33]
		Na *et al.*	2009	[34]
		Mahmoudi *et al.*	2011	[36]
		Jordan *et al.*	2001	[37]
		Pankhurst *et al.*	2003	[38]
		Varadan *et al.*	2008	[40]
	**Mixing with Beads/Chaining**	Martin *et al.*	2009	[42]
		Owen *et al.*	2013	[43]
		Raman *et al.*	2012	[44]
		Dreyfus *et al.*	2009	[45]
**Sensing**	**Magnetic Particle Sensing**	Eickenberg *et al.*	2013	[26]
		Takamura *et al.*	2015	[27]

## 2. Magnetic Bead Properties and Synthesis

### 2.1. Superparamagnetic Nanoparticles in Microfluidics

Let us now have a more detailed view at the physical phenomenon called superparamagnetism. Superparamagnetism is a type of magnetism occurring in sufficiently small ferromagnetic or ferrimagnetic nanoparticles. Normally, ferromagnetic or ferrimagnetic material undergoes a transition to a paramagnetic state above its Curie temperature. This form of magnetism describes the behavior, when materials are attracted by an external magnetic field and an internal field is induced in the material, which is oriented along the direction of the applied field. Paramagnetic materials feature a relative magnetic permeability greater than or equal to 1. The magnetic moment induced in the material by the applied field is rather weak. Superparamagnetism is different to paramagnetism with respect to the transition temperature since it occurs below the material’s Curie temperature.

Furthermore, nanoparticles underlie a magnetic anisotropy: the magnetic properties depend on the direction. This means, the magnetic moment tends to align along the easy axis of the material, while in case of an isotropic material, no preferential direction for its magnetic moment is found in the absence of an external field. Because of the nanoparticle’s magnetic anisotropy, the magnetic moment usually has two orientations, arranged antiparallel to each other along the direction of the easy axis. These two orientations are separated by an energy barrier. At finite temperature, there is a certain probability for the magnetization to flip and reverse its direction. The direction of magnetization randomly flips between these two states with a typical relaxation time, called Néel relaxation time τ_N_. This characteristic time exponentially depends on the term (*KV*/k_B_*T*) as given by the Néel-Arrhenius equation [14]:

τ_N_ = τ_0_ × exp(*KV*/k_B_*T*) (1)

with the material specific attempt period τ_0_ (typically in the order of 1–100 ps), the magnetic anisotropy factor *K* (J/m³), the sample volume *V*, the Boltzmann constant k_B_ ≈ 1.38 × 10*^−^*^23^ J/K, and the absolute temperature *T*. The product *KV* is the energy barrier mentioned above and needs to be overcome for the magnetic moment to flip. The term k_B_*T* describes the thermal energy of the sample.

**Figure 1:**
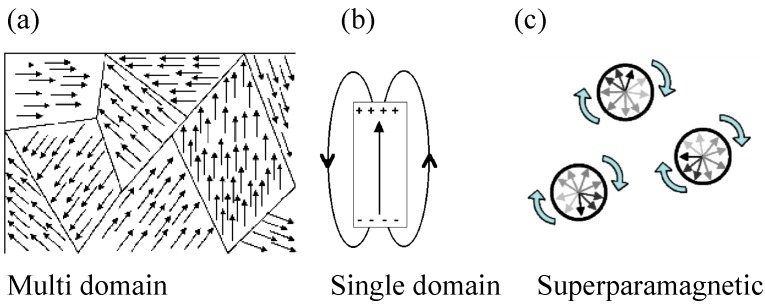
Magnetic behavior of a magnetic sample featuring (**a**) a multidomain structure, (**b**) a single-domain, and (**c**) superparamagnetic properties (adapted from [15]).

Ferromagnetic or ferrimagnetic nanoparticles can be magnetized by an external magnetic field similar to a conventional paramagnet. In the absence of an external magnetic field, the average magnetization is zero. Superparamagnetic properties turn out to be ideal for sample manipulation, since an immobilization and a directed transport by means of external magnetic fields are possible and the sample bound to the magnetic beads can easily be released from the immobilized state by simply switching off the magnetic field and thus discontinuing the magnetic interaction. An additional advantage of superparamagnetism is that the superparamagnetic beads do not agglomerate after switching off or removing the external magnetic field, since no permanent magnetization remains. Figure 1 sketches the magnetic behavior of a multidomain structure, a single-domain particle, and a superparamagnetic sample. According to the Néel-Arrhenius Equation (1), the magnetic behavior exponentially depends on the magnetic volume *V* (*i.e.*, the particle size), the magnetic anisotropy factor *K* (which is material specific), and the ambient temperature *T* of the sample. The magnetic moment spontaneously flips with the Néel relaxation τ_N_ time as characteristic period.

**Figure 2:**
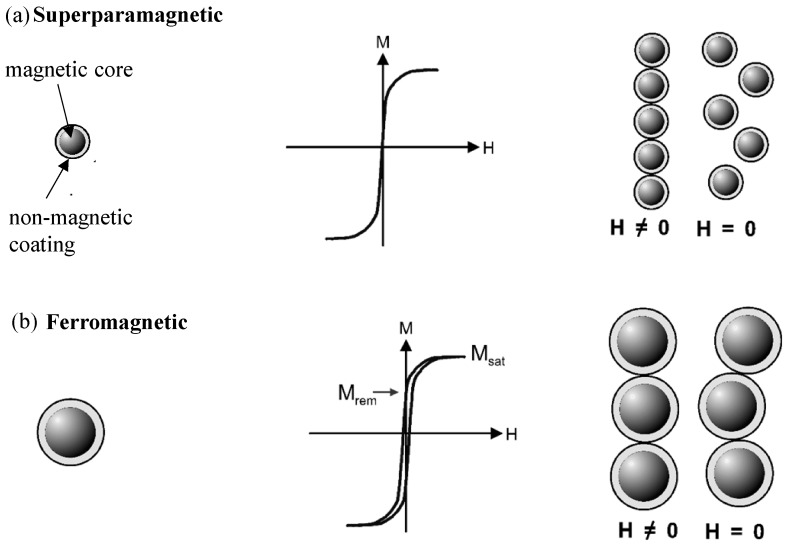
Comparison of superparamagnetic and ferromagnetic particles, from left to right: (**a**) Schematic diagram of a spherical magnetic nanoparticle with a non-magnetic coating (**left**), magnetization loop of an ensemble of nanosize superparamagnetic particles (**center**), and nanoparticle superstructure exposed to a magnetic field ***H*** and decomposition of the superstructure after field removal (**right**). (**b**) Schematic diagram of a spherical magnetic microparticle (**left**), magnetization loop of an ensemble of ferromagnetic particles (**center**), and superstructure in a magnetic field keeping a remnant moment and chain-like superstructure after field removal (**right**) (adapted from [4]).

Depending on their size and the magnetic content, which typically consists of magnetite (Fe_3_O_4_) or maghemite (mostly in the face-centered cubic crystal modification γ-Fe_2_O_3_), magnetic nanoparticles feature a superparamagnetic or a ferromagnetic behavior. Figure 2 compares superparamagnetic and ferromagnetic behavior of spherical magnetic nanoparticles and microparticles. Figure 2a left sketches a spherical nanoparticle with a magnetic core and a non-magnetic coating featuring superparamagnetic properties. The corresponding magnetization loop in the center shows the characteristic magnetic properties of an ensemble of superparamagnetic nanoparticles: neither a hysteresis, nor a remnant magnetization are found proven by the zero-crossing of the curve. On the right in Figure 2a, a schematic diagram of a nanoparticle superstructure in the presence of a magnetic field ***H*** is shown. When the magnetic field is switched off, the superstructure decomposes into single particles. This is attributed to the absence of a remnant magnetization. No interaction occurs between the single particles without an external magnetic field. Figure 2b represents on the left a schematic diagram of a spherical magnetic microparticle. The corresponding schematic magnetization loop in the center illustrates the magnetic characteristics of an ensemble of such ferromagnetic particles featuring a magnetic hysteresis. The remnant magnetization *M*_rem_ and the saturation magnetization *M*_sat_ are indicated. In the presence of a magnetic field ***H***, a microparticle superstructure is created with a chain-like appearance like illustrated in the right picture of Figure 2b. When the magnetic field ***H*** is removed, the microparticles keep a remnant magnetic moment. As a consequence, the superstructure does not decompose as in case of superparamagnetic particles illustrated in Figure 2a in the right picture, since the magnetic interaction is still present [4].

Single domain magnetic particles become superparamagnetic, when their magnetic energy described by the magnetic anisotropy factor times the particle volume *KV*, known from Equation (1), is smaller than about ten times the thermal energy k_B_*T*: *KV* ≤ 10k_B_*T*. At room temperature, the maximum radius for a superparamagnetic spherical particle made of iron is 6 nm [4]. For other magnetic materials, the limit for exhibiting superparamagnetic behavior is in the range of 3–50 nm. Beside the particle size, the applied field and the ambient temperature of the sample play a role. The magnitude of magnetization is proportional to the applied magnetic field ***H*** = ***B***/µ_0_. This proportionality is reduced with rising temperature. For a constant field, the magnetization is approximately inversely proportional to the temperature. This behavior is described by Curie’s law (see for example [15]):

***M*** = *C* × ***B***/*T* (2)

with the magnetization ***M***, the magnetic induction ***B***, the absolute temperature *T*, and *C* presenting a material-specific constant, called Curie constant dedicated to Marie Curie. The increase in temperature leads to an increase of the thermal agitation of the atoms. This destroys the alignment of the atoms along the magnetic field. Hence, the magnetization of the magnetic material decreases.

Magnetic particles in a fluid exposed to an external magnetic field are subject to various forces. The most important ones are the hydrodynamic drag or viscous force, and the magnetic force exerted on the particles by the external magnetic field. To be able to manipulate the particles by means of the magnetic field, the magnetic forces have to exceed the forces caused by the viscous flow of the fluid. This has to be considered when designing a respective microfluidic system. The viscous force ***F***_v_ can be calculated following Equation (3) based on Stoke’s law:

***F***_v_ = 6π*r*η × Δ***v*** (3)

with the particle radius *r* and the dynamic viscosity η. The viscous drag force is a consequence of the velocity difference Δ***v*** between the particle velocity and the velocity of the surrounding fluid. The magnetic force ***F***_m_ acting on superparamagnetic beads in an external magnetic field ***H*** = ***B***/µ_0_ is proportional to the sample volume *V* and the magnetic susceptibility χ_m_. The susceptibility determines the magnetic behavior of a magnetic material in a magnetic field, whether is attracted into the field or repelled out of the field. Superparamagnetic particles feature a much higher magnetic susceptibility than paramagnets. Furthermore, the magnetic force ***F***_m_ depends on the field gradient ∇***H*** = ∇***B***/µ_0_ as given by Equation (4):

***F***_m_ = *V* × χ_m_ × µ_0_^−1^(***B*** × ∇)***B*** (4)

Equalizing the viscous force from Equation (3) and the magnetic force from Equation (4) by ***F***_v_ = ***F***_m_ shows that the velocity difference ∆***v*** between magnetic beads and the fluid is proportional to the magnetic susceptibility χ_m_ and the term (***B*** × ∇)***B*** and thus the second order of the field gradient. This means, the effective velocity depends on the magnetic material of the bead by χ_m_ and the strength and configuration of the applied magnetic field (see also [4,9]).

### 2.2. Magnetic Bead Fabrication and Detection

The following chapter presents approaches of nanoparticle and magnetic bead fabrication and then points to the related challenges. With spreading application of magnetic beads, the ability to adjust the properties of magnetic nanoparticles as bead precursors becomes more important. Design criteria like size, coating, and surface functionalization have to be properly adjusted and fulfilled.

Veiseh *et al*. present design parameters affecting the performance of magnetic nanoparticles *in vivo* covering nanoparticle surface modification and physicochemical properties [16]. Park *et al.* demonstrate basics for the fabrication of magnetic nanoparticles with tunable size, magnetic properties, and surface binding capabilities [17]. The fabrication of gold-coated magnetic nanoparticles with a specific core-shell nanostructure for protein immobilization was shown. Iron oxide (Fe_2_O_3_ and Fe_3_O_4_) nanoparticles with a controllable size within the range 5–100 nm, exhibiting a high monodispersity could be synthesized. Finally, the group of Park demonstrated the surface protein-binding reactivity and separation exemplarily for a bioassay by means of a magnetic field. Zhao *et al.* describe the synthesis of mesoporous silica shell nanospheres with a magnetic core, featuring a uniform particle diameter of about 270 nm [18]. Due to its mesoporous structure, the silica shell provides a high surface area for sample attachment suitable for drug delivery and separation applications. The efficiency of the nanosphere binding capability is exemplarily shown by the capture and release of ibuprofen. Kim *et al.* prepared silica nanospheres containing iron oxide (Fe_3_O_4_) with a gold coating for magnetic resonance imaging and cancer therapy. Via a polyethylene glycol (PEG) linker, a specific antibody was attached to the nanosphere surface, enabling the specific capture of cancer cells [19]. Ito *et al.* present magnetic force-based tissue engineering [20]. The basic idea is an uptake of magnetite nanoparticles by the respective target cells. Two types of tissue were investigated: urinary tissue and vascular tissue. To demonstrate the proof-of-concept, tubular tissue structures were created by means of magnetic forces.

**Figure 3:**
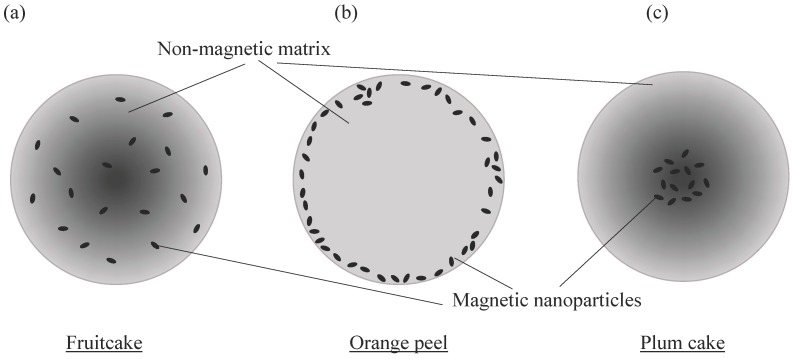
Variations of nanoparticle embedding in a non-magnetic spherical matrix creating superparamagnetic beads, from left to right: (**a**) fruitcake, (**b**) orange peel (cross-section), and (**c**) plum cake like distribution of magnetic nanoparticles.

Three forms for the embedding of magnetic nanoparticles in a matrix to create paramagnetic micro-sized beads is introduced by Thanh [21]. The variations are named after their appearance “fruitcake” (magnetic nanoparticles uniformly distributed in the matrix), “orange peel” (particles arranged close to the surface of the bead), and “plum cake” (particles concentrated in the bead center). Figure 3 schematically visualizes these three types of nanoparticle embedding in a non-magnetic matrix.

The fabrication of magnetic beads has been investigated and understood to a large extent. Nonetheless, there are still challenges to be met related to the synthesis of magnetic nanoparticles. An essential task is to achieve a uniform size and shape. In addition, uniformity with respect to the magnetic and chemical properties of the fabricated particles is a precondition not only for the reproducibility of scientific results, but also for commercialization. Furthermore, magnetic particles used in biomedical applications should be water-dispersible. This means they should feature a hydrophilic surface coating. Their surface should lend itself for a sample-specific functionalization, which has to be biocompatible in most applications.

A facile one-step reverse precipitation method to synthesize water-dispersible, biocompatible, and carboxylate-functionalized superparamagnetic magnetite (Fe_3_O_4_) nanoparticles utilizing biocompatible sodium citrate salt was used by Jing *et al.* [22]. Transmission electron microscopy (TEM), X-ray diffraction (XRD), Fourier transform infrared spectroscopy (FTIR), zeta potential measurements, and dynamic light scattering (DLS) were applied for the characterization of the as-prepared magnetite nanoparticles. By changing the sodium citrate concentration within the range of 25 to 125 mM, the size of the magnetite nanoparticles could be tuned from 27 ± 3.8 to 4.8 ± 1.9 nm. It was found by Jing *et al.*, that the sodium citrate concentration had an influence on the water-dispersible stability of the fabricated magnetite nanoparticles. This effect was attributed to electrostatic repulsion effects among the particles.

The discussion of the optimum particle size for magnetic nanoparticles by Colombo *et al.* has been a point of controversy. A compromise has to be found between a large magnetic moment on the one hand, which is related to bigger particles, and biocompatibility on the other hand, which becomes critical when the particles are too small [23]. Following the authors, the optimum size for ferritic nanoparticles is found between 10 nm, corresponding to the superparamagnetic size limit for the considered material at room temperature, and about 70 nm, which is the critical domain size. The properties of various magnetic nanoparticles are compared and surface functionalization methods including their applicability for bioconjugation are evaluated.

Nowadays, a huge variety of magnetic beads featuring a large diversity for different applications is commercially available. Just to name a few: Dynabeads^®^ Magnetic beads provided by Invitrogen, Estapor^®^ SuperParamagnetic Microspheres and PureProteome™ Magnetic Beads by Merck Millipore, BcMag™ by Bioclone Inc., ProMag™ and BioMag^®^ from Bangslabs, SupraMag™ by Polymicrospheres Inc., TurboBeads^®^ by Turbobeads Llc., and SPHERO™ Polystyrene Magnetic Particles by Spherotech. Other companies like Sigma-Aldrich or Thermo Scientific, Microparticles, and Microspheres-Nanospheres offer superparamagnetic beads as well. The primary use of these commercial beads is binding, purification, and magnetic separation of biomolecules comprising proteins, cells, DNA fragments, and other biomolecules such as nucleic acids, enzymes, antibodies or bacteria. Among the broad spectrum of purchasable beads, also digital barcoded beads are provided. Maxwell Sensors Inc. holds a patent for the fabrication of magnetic beads with a digital code, which are designed to significantly improve the isolation and identification capacity of molecular diagnostics. This is particularly relevant for high-throughput applications or multiplexed assays. Digital optical barcodes are generated lithographically on the magnetic beads, whose surface coatings are able to specifically bind nucleic acids, proteins or other biomolecules of interest. Meanwhile, various digital magnetic beads for molecular diagnostics and life sciences are commercially available [24,25].

A permanent challenge with respect to the detection is the availability of adequate detection tools and methods, since the magnetic detection signal is scaling with the magnetic volume in the sample, which is very small in case of magnetic beads. Superconducting QUantum Interference Devices (SQUIDs) are commonly used for the high-precision measurement of the extremely small magnetic field changes produced by magnetic nanoparticles. In addition to SQUIDs, magnetoresistive (MR) sensors or Hall sensors are used. A tabular overview of the methods is included in the book by Thanh [21].

However, the ability for the detection of a field strength close to zero does not ensure that all particles in the sample are registered: for example, superparamagnetic beads utilized as labels in immunoassays suffer from the fact that a high number of the beads pass the sensing area without being detected. Various solutions employing magnetic forces, ultrasonic standing waves, and even hydrodynamic effects have been developed to cope with this issue. Eickenberg *et al.* give a more detailed description on these approaches and propose a new sensor concept circumventing the issues indicated before. This novel concept is based on the granular giant-magnetoresistance (GMR) effect found in gels containing magnetic nanoparticles. The principle is as follows: Antibodies on the gel surface bind to antigens on the surface of the beads in the fluidic sample. The magnetic stray field of the beads changes the resistance of the gel through the granular GMR effect, enabling the detection and quantification of the bound beads [26]. In a review on magnetic particle sensing, Takamura *et al.* report on an approach for the detection of sub-200 nm magnetic particles via columnar particle arrangements [27]. Llandro *et al.* describe the physical concepts of various sensor types used to detect magnetic labels in the application field of magnetic biosensors [28]. The biocompatibility of magnetic substrates is discussed as well in this paper. This overview shows that various methods and tools are available nowadays, but the detection of extremely weak fields generated but such small-size particles is still a challenge, stimulating further and new developments in magnetic detection.

### 2.3. Multifunctional Hybrid Nanoparticles

Colloidal nanocrystals are used in various fields of science. These include biology, medicine, the development of new diagnostic methods and tools, drug delivery, imaging, up to physics and engineering and new equipment for energy conversion and storage. They may be equipped with multifunctional properties to extend their applicability or make their use more efficient. The control of the properties of such nanocrystals is carried out via the setting of their size, shape, and composition during the chemical synthesis as well as careful selection of their organic coating. Chen *et al.* report on approaches to control the shape and morphology of platinum nanocrystals to improve their catalytic activity and electrocatalytic properties [29].

Scientists at the Institute Nanoscience in Lecce, Italy, and the Italian Institute of Technology in Genoa, Italy, synthesized trifunctional polymers nanobeads from a mixture of magnetic nanoparticles (responsible for the magnetic properties), quantum dots (serving for luminescent features of the beads), and an amphiphilic polymer [30]. An increasing luminescence of the nanobeads with increasing quantum dot concentration could be detected. This is important for imaging applications. The distribution of magnetic nanoparticles within the beads and thus the speed of the magnetic response of the system in a magnetic field could be adjusted by adjusting the destabilizing agent. As destabilizing agent, water or acetonitrile was used. The third function beside magnetic properties and the luminescence was provided by a specific surface coating. By coating the beads with folic acid, cancer cells overexpressing folate receptors could be targeted. The increased intake of the nanoparticles by cancer cells was ascribed to an over-expression of folic acid receptors.

**Figure 4:**
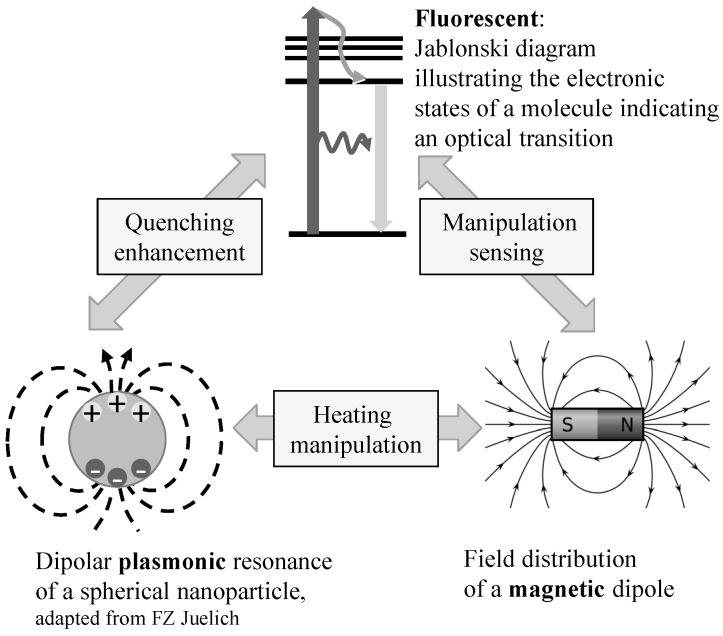
Properties for preparing bi- and trifunctional nano-objects and their bilateral interaction (adapted from [31]).

Bigall *et al.* present colloidal nanocrystals with hybrid functions that combine at least two of the following characteristics: fluorescent, magnetic, and plasmonic [31]. Surface plasmons are delocalized electron oscillations found at the interface between two materials, generated from the interaction of light with certain dielectric materials. They have an impact on the optical properties of the material. The possibility of providing nanocrystals with hybrid functionality, as illustrated in Figure 4, opens up a wide field of applications in biotechnology and medicine.

## 3. Magnetic Bead Applications

### 3.1. Introduction to the Use of Magnetic Beads

The use of magnetic beads can be grouped in two main applications fields: magnetic beads used as substrate carrying the sample of interest and their application as detection label attached to the sample. In the review paper of Gijs *et al.* from 2004 describing the state-of-the-art in the area of magnetic bead handling and manipulation, the focus is set on the use of magnetic particles in microfluidic systems [4]. Beside fundamental physical aspects of magnetic actuation, the variety of magnetic bead applications is presented and pros and cons related to the use of magnetic beads are illustrated.

Magnetic beads can be manipulated either by permanent magnets or electromagnets. Static fields are used for sample immobilization and field gradients for the transport of the sample attached to a magnetic bead. This can be accomplished independent of any other chemical, biological, or microfluidic processes. In addition, magnetic labels do not bleach, like fluorescent labels do. This opens up a wide spectrum of analysis and manipulation on microchip level. The spectrum includes immunoassays (see next chapter), cell manipulation and cellular-specific targeting [21], DNA extraction [32], magnetic resonance imaging (MRI) [33,34,35], targeted medication [35], and hyperthermia therapies [33,37] in medical applications [30,38]. In particular, magnetic bead-based immunoassays featuring a microfluidic format have attracted much attention. Already in 1998, Sinclair recommended the use of magnetic particles for isolation and purification methods in biological applications [2]. However, the establishment of magnetic bead in this field of application required some more years since then.

As mentioned in the introduction, the use of magnetic beads is not restricted to biomedical applications at all. In principle, the beads lend themselves for the separation or manipulation of any kind of sample, which can be bound to a magnetic bead surface. For instance, magnetic beads were used for the capture of colloidal nanoparticles of the platinum group [13]. This was carried out in view of a reuse of the metallic nanoparticles serving as catalyst for various small-scale synthetic reactions. The avidin-biotin system was employed to link the magnetic beads to platinum nanoparticles. In fact, the gold-thiol system works much better, but the chemical binding is too strong to allow for a recycling (requiring a detachment of the metallic nanoparticles from the beads after separation). A separation of the metallic nanoparticles attached to the magnetic beads with common external magnetic field strengths was proven. First investigations indicated a successful detachment of the platinum nanoparticles from the magnetic beads after the separation from a mixture. This study on catalyst recovery by magnetic separation is a step to discover new markets for magnetic beads.

### 3.2. Magnetic Beads in Nanomedicine

An immunoassay is a biochemical test serving for biomolecule detection and quantification. In an immunoassay, the detection and quantification of molecular particles or antigens is based on the specific interaction and formation of a complex consisting of an antibody and an antigen. Depending on the configuration of the assay, either the antigen or the antibody is detected. Usually, a fluorescent marker is used for the detection of the complex, which is formed during the assay [39]. In such an immunoassay, magnetic beads can be used as a mobile substrate or as a marker. The basics of immunoassays with the focus on magnetic particles used as label are described by Thanh [21].

**Figure 5:**
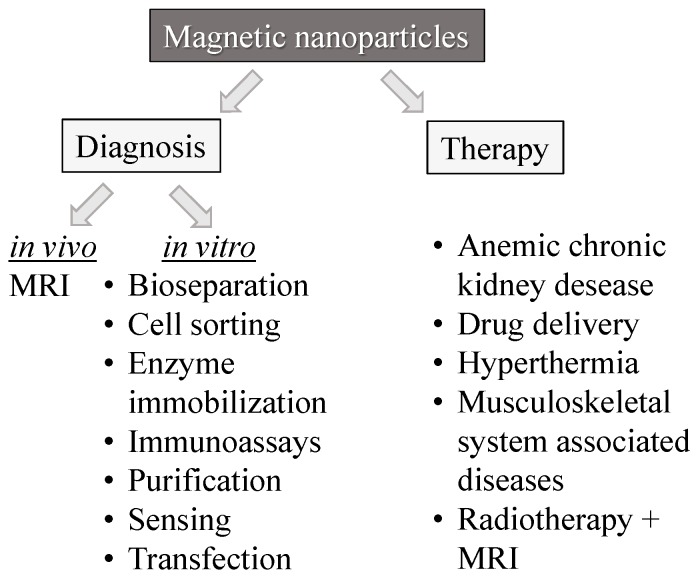
Magnetic nanoparticles in biomedicine: diagnosis and therapy (adapted from [40]).

The book by Varadan *et al.* covers a fundamental description of methods for the prevention, diagnosis, and treatment of diseases offered by nanomedicine [40]. The book includes an introduction to the physical and chemical principles of magnetic nanomaterials and presents an overview of biomedical applications of functional magnetic material. Figure 5 provides an impression of the diversity and potential of magnetic nanomaterials for diagnosis and therapy in biomedicine.

According to Figure 5, magnetic nanoparticles find their biomedical applications in the fields of diagnosis and therapy. Regarding diagnosis needs, they may serve as contrast agents *in vivo* in MRI as well as in various *in vitro* applications covering sample separation, immobilization, and immune-reactions. Additionally, magnetic nanoparticles are employed for the therapy of a number of several severe human diseases.

**Figure 6:**
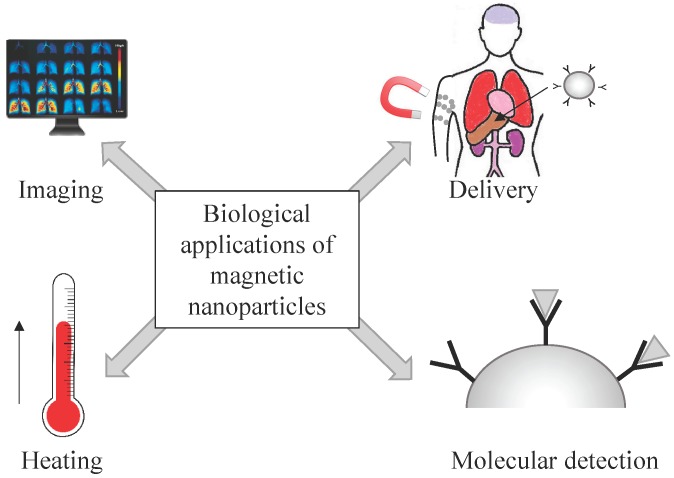
Overview of biological applications employing magnetic nanoparticles (adapted from [22]).

Li *et al.* discuss the promising capability of nanoparticles and nanotubes in medical imaging, and diagnostics as well as in therapeutics and analyze the issue of biocompatibility and toxicity in their review article [41]. A critical article on the use of magnetic nanoparticles in nanomedicine, considering also possible risks for humans, is provided by Colombo *et al.* [23]. The authors describe *in vitro* and *in vivo* applications of magnetic colloidal nanoparticles. The future role of magnetic nanoparticles with respect to other functional nanoparticles is discussed as well as the possible integration with existing biotechnological methods. Issues related to the outcome of the particles in the body are pointed out in order to address the remaining challenges for an extended use of magnetic nanoparticles in medicine. Figure 6 visualizes some basic applications for magnetic nanoparticles in biomedicine, which are found in Figure 5: their use as contrast agents in MRI, drug delivery, hyperthermia, and sensing or detection.

On *in vitro* level, the systems employed for nanomedicine are microfluidic systems. The magnetic beads or functionalized nanoparticles are distributed in a liquid carrier medium and guided through a system consisting of channels for guiding and transport, and chambers for mixing and chemical reactions and proper inlets/outlets. Main advantages of microfluidic systems are:

(i) Significant reduction of sample and reagents (even down to the picoliter scale);
(ii) Fast reaction times (when the molecular diffusion length matches the channel dimensions);
(iii) Large surface-to-volume ratio (enabling efficient binding processes of the sample).

These advantages related to microfluidic systems make those useful tools for innovative developments in biomedical sciences demanding minimal or even non-invasive, highly efficient operations.

### 3.3. Magnetic Chaining

In addition to the use of magnetic beads as substrate or label, they can be considered as an innovative tool for mixing purposes in microfluidics. Creating magnetic bead chains, rotating in an alternating magnetic field, is a simple method to achieve effective mixing of different fluids in a microfluidic system. Furthermore, single magnetic beads or magnetic bead clusters attached to the inner surface of a microfluidic system might create vortices in the channel, serving as passive mixing structures.

Especially small confined geometries, such as microfluidic channels, require improved methods of fluid mixing since the present low Reynolds numbers impede the induction of turbulences. When a magnetic field is applied to a microstructure containing a fluid with dispersed magnetic beads, the beads become magnetized and align with their neighbors. This creates columnar or chain-like structures. The chains are formed in the direction of the magnetic field, but the movement is along the magnetic field gradient direction.

There have been numerous attempts to address this issue with magnetic fields. Martin *et al.* achieve bead mixing with a triaxial magnetic field, called vortex field. The vortex field is composed of a rotating magnetic field in a horizontal plane, superposed by a DC field applied normal to the rotating field [42]. Owen *et al.* achieve an effective mixing of superparamagnetic beads in a fluid-filled microchannel via an external rotating permanent magnet on a short distance [43]. A mesoscale computational method is used to model the bead dynamics and to determine the variables which are supposed to have an impact on the mixing: flow rate, bead rotation speed, and bead number density, respectively.

It should be mentioned, that the use of magnetic bead columnar arrangements or chains is not only restricted to mixing: Raman *et al.* used the chaining effect for the determination of the orientation in copolymer nanodomains through the alignment and chaining of superparamagnetic nanoparticles in a magnetic field [44]. Dreyfus *et al.* accomplished the measurement of colloidal forces with the magnetic chaining technique [45], while Takamura *et al.* accomplish magnetic particle detection based on magnetic bead chains in rotating magnetic fields [27].

### 3.4. Biomolecule Detection—Multiplex vs. Singleplex Approaches

This chapter provides an overview of standard and novel technologies for biomarker detection and quantification. Multiplex as well as singleplex approaches based on superparamagnetic beads are presented as well as magnetic bead-based cell sorting methods. Multiplex assays simultaneously measure multiple analytes in a single run, while singleplex assays target only one analyte. Exemplarily, cytokines are considered as a prominent category of biomolecules. Cytokines are small proteins (about 5–20 kDa) that play an important role in cell signaling. They mediate a variety of biological activities, including cytotoxic and immunomodulatory effects. Cytotoxicity describes the property of being toxic to cells, immunomodulation the capability of modifying or regulating immune functions of an organism. Their presence of cytokines at elevated levels is associated with inflammation, disease progress or even septic shock [46]. Owing to the significant diagnostic value, it is essential to quantify the cytokines in an accurate and timely manner in order to understand the origin of diseases and help in designing of treatment strategies.

Nowadays, various cytokine assays are available including flow cytometry, mRNA based assays, Enzyme-Linked Immuno SPOT (ELISPOT) assays, IntraCytoplasmic Cytokine (ICC) staining, and magnetic bead-based immunoassays. Conventionally, these assays are multiplex approaches. Among these assays, the magnetic bead approach allows a rapid and efficient capture, separation, and quantification of specific biomolecules. Conventional magnetic bead assays are performed using standard 96-well plates in an Enzyme-Linked Immune-Sorbent Assay (ELISA)-based format. Nonetheless, this approach is not efficient for the detection of a single biomarker, since ELISA is designed for the parallel detection of various analytes in a high-throughput multiplex modus. The Luminex^®^ xMAP^®^ technology (Luminex® Corp., Austin, TX, USA) is set up to measure multiple protein targets in a sample simultaneously. Both, magnetic and polystyrene (*i.e.*, non-magnetic) reagent kits are commercially available. Any of the methods listed before are multiplex approaches missing an effective method for the needs of singleplex applications. To fill this gap, an integrated magneto-microfluidic device was designed based on a magnetic bead-based immunoassay for the capture and quantitation of a single cytokine. This microfluidic lab-on-chip setup provides an efficient way to measure single biomarkers as a competitive alternative to multiplex approaches [12].

Conventionally, biological cells in a liquid carrier medium are sorted with the Magnetic activated cell sorting (MACS^®^) technology (Miltenyi Biotec GmbH, Bergisch Gladbach, Germany). The MACS^®^ technology is also known as the gold standard in cell separation and was developed in 1990 by Miltenyi *et al.* [47]. This method relies on the attachment of cells to specifically-coated superparamagnetic particles and the subsequent manipulation by means of a magnetic field gradient. A competitive approach to the MACS^®^ technology is provided by Invitrogen. The Magnetic Particle Concentrator MPC^®^ (Invitrogen™, Thermo Fisher Scientific Corp., Waltham, MA, USA) provided by this company is used to bind Dynabeads^®^ (Thermo Fisher Scientific Corp.), an in-house development of superparamagnetic particles, to the target molecules such as cells, proteins or nucleic acids. The system MPC^®^-S has been specifically designed for microbiological applications and was validated with selected Dynabeads^®^ for the enrichment of *Escherichia coli (E. coli)* O157:H7, salmonella, and several other bacterial strains. The system can be used for food and water analyzes as well. An alternative supplier for magnetic particle-based cell separation is the company R&D Systems. They offer MagCellect™ (R&D Systems Inc., Minneapolis, MN, USA) ferrofluid conjugates in collaboration with Immunicon Corp. [48]. The EasySep™ (StemCell Technologies Inc., Vancouver, BC, Canada) immunomagnetic cell isolation platform provided by StemCell Technologies Inc. manages the separation of human cell populations within only 25 min. [49]. Immunicon Corp. is considered as an expert not only in the area of cell-based research but also for the development and commercialization of diagnostic products with a focus on cancer diseases. With the technologies developed at Immunicon Corp., tumor cells circulating in the bloodstream of a patient can be detected and quantified on a very low concentration level. The CellSearch^®^ (Janssen Diagnostics LLC, South Raritan, NJ, USA) Circulating Tumor Cell Kit enables the immunomagnetic selection, identification, and quantification of circulating tumor cell with an epithelial origin from the whole blood [50]. The Cell Separation Magnet system by BD Biosciences offers positive and negative selection as well as the separation of leukocytes labeled with superparamagnetic BD Imag™ (Becton Dickinson, Franklin Lakes, NJ, USA) particles [51]. A comprehensive overview of commercial magnetic cell separation devices is given by Zborowski and Chalmes [52].

## 4. Magnetic Manipulation—Practical Aspects

In the early stages, biochemical sample mixtures were separated by batch processes, including filtration, centrifugation, chromatography, and electrophoresis. However, when microfluidics appeared on the scene and achieved more and more interest in research groups all over the world, continuous flow separation methods in microfluidic devices were developed as an alternative to the batch processes. A large variety of separation methods has been reported, comprising miniaturized models of large-scale methods as well as such only applicable in microfluidic flow regimes. A description and comparison of microfluidic separation processes via forces generated by external fields is provided by Pamme [6,53].

**Figure 7:**
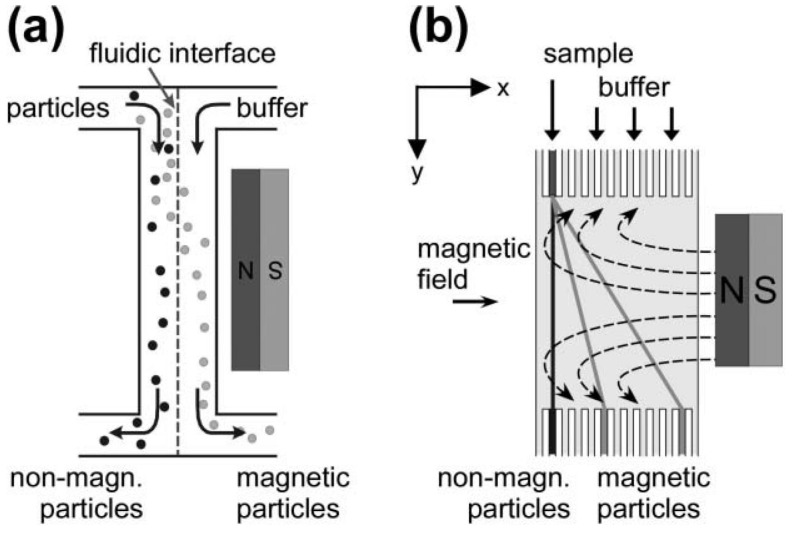
Magnetic separation from a continuous flow: applying a magnetic field perpendicular to the direction of the fluidic flow results in a deflection of magnetic particles or magnetically labeled molecules. (**a**) Separation of non-magnetic particles from magnetic particles via two outlets; and (**b**) system with multiple outlets (reprinted with permission from [6]).

For practical use, it is desirable to separate the sample from a continuous flow and not from a stationary state. This way, a higher throughput can be achieved and the sample and waste are separated in real time. Various approaches for continuous flow separations have been suggested. For example, magnetic particles can be deflected from a continuous fluidic flow on-chip by a magnetic field, arranged perpendicular to the direction of the flow. In this case, the degree of deflection depends on not only on the magnetic field strength, but also on the magnetic susceptibility of the particles in the sample. This magnetophoresis-based separation approach may be applied for sorting of magnetic microparticles and nanoparticles after fabrication or cells equipped with a magnetic label. Figure 7 visualizes the principle of magnetic separation from a continuous flow in a microfluidic system by deflection caused by a magnetic field arranged perpendicular to the flow.

Microfluidic separation methods are characterized by continuous injection, real-time monitoring and continuous collection. A basic feature of continuous-flow separation methods is the deflection of sample components from the main direction of flow. This is accomplished either by a force, provided by an external electric, magnetic, acoustic or optical field, or by arranging obstacles in the laminar flow deflecting certain sample components spatially. The attachment of a magnetic component like a magnetic bead is required in most cases, since particularly biomolecules are not susceptible to magnetic manipulation with only very few exceptions (such as erythrocytes with a certain iron content and magnetotactic bacteria). When magnetic forces are applied for particle separation, neither ionic nor surface charges nor the pH value may have an influence on the susceptibility of manipulation. Furthermore, magnetic manipulations mostly do not modify or damage the sample and are thus well suited for biomolecules. Magnetic separation thus presents a versatile tool in the field of microfluidics.

## 5. Conclusions

The proposal of Micro Total Analysis Systems (µTAS), also known as lab-on-a-chip (LoC) systems, initiated an explosive increase in the research activities to develop miniaturized systems allowing multifold operations on a single chip. All functions of a macroscopic laboratory are intended to be integrated on a credit card-sized device. The LoC approach provides tools for the study of biological interactions down to the cellular level and molecular scale and provides a huge potential for applications in life sciences and related fields of research. Main advances of a lab on a chip are the miniaturization and transportability, which lends themselves to use in developing countries or in case of medical emergency. Furthermore, the sample volume and the consumption of reagents are reduced to a minimum. As a consequence, costs are reduced significantly as well. Fast reaction times and waste volumes close to zero provide a competitive approach to standard laboratory analyses methods. In practical applications, systems with low fluid volumes are processed to achieve multiplexing, automation, and high-throughput screening [54].

The application fields for magnetic beads extensively discovered so far range from immunoassays to cell manipulation, DNA extraction, magnetic resonance imaging, and hyperthermia therapies to the integration in colloidal nanocrystals. Especially in the area of miniaturized microfluidic systems, magnetic particles and nanomaterials play a dominant role. The unrivalled advantage of such superparamagnetic particles is the possibility to switch off the magnetic interaction within the sample or between the sample and the external field by just removing the external magnetic field. In addition, magnetic manipulation approaches do not damage or modify the sample, which is important especially in biomedical applications. Furthermore, neither ionic or surface charges nor the pH value may deteriorate the manipulation susceptibility.

Still an issue is the availability of adequate tools for the detection and quantification, which is an everlasting challenge in case of magnetic material with such a small magnetic content and physical size like magnetic beads have. Next, there are still no consistent standards in the microfluidic community for fittings, interfaces, or infrastructure in terms of plug-and-play components or toolboxes. In most cases, own solutions are applied. The *Microfluidics Consortium*, organized by the Centre for Business Innovation in Cambridge, UK, intends to identify and promote precursors to standards (e.g., connectors and chip holders) [55]. Recently, an online discussion was released, proposing ISO standards for microfluidic connectors [56].

The time scale from the level of development to industrial use and acceptance is quite long. Maybe, one bottleneck is the high multidisciplinary of microfluidics, especially in combination with the use of magnetic beads intersecting engineering, physics, bio-chemistry, nanotechnology, and biotechnology. For example, scientific and practical knowledge is required on a high level in inorganic chemistry (required for magnetic bead preparation) and in biochemistry as well as in medical sciences (to equip the beads with an adequate functionalization matching the sample needs). Not least, basic physical principles of nanomagnetism and magnetic materials should be familiar. This requires not only well-trained staff at least during the development phase, but also a multidisciplinary team. Another barrier might be related to the manufacturing of the microfluidic chips: while the fabrication of LoC devices is quite straight forward, the master mold fabrication normally requires a cleanroom facility. This might be another inhibition, especially for small and medium-sized enterprises to step into microfluidics.

Nonetheless, an ongoing use and a prosperous future is predicted for microfluidic systems in general and notably for the material class of magnetic beads, which can be considered as a magic bullet in the sense of a miracle cure for various medical tasks or—in a figurative sense—as magic bullets. The denotation “magic” is used here due to their unique or even hybrid properties in combination with their versatile use and “bullet” simply according to their spherical appearance. After extensive investigations in bioanalysis and biomedical sciences, it is time to discover new markets, since the use of magnetic beads is not restricted to biomolecules. A promising field is catalyst recovery. Only very few studies have been carried out so far in the field of magnetic catalyst recovery, but those delivered promising results. Catalyst recovery or spoken more general *Green Microfluidics*, the recovery and recycling of chemicals and reactants after their use and the preparation for reuse might be an auspicious market gap for magnetic beads.
